# A Randomized Placebo-Controlled *N*-of-1 Trial: The Effect of Proton Pump Inhibitor in the Management of Gastroesophageal Reflux Disease

**DOI:** 10.1155/2019/3926051

**Published:** 2019-12-12

**Authors:** Fernando Sierra-Arango, D. M. Castaño, Jennifer D. Forero, Erika D. Pérez-Riveros, Gerardo Ardila Duarte, Maria L. Botero, Andres Cárdenas, Jose De la Hoz-Valle

**Affiliations:** ^1^Gastroenterology and Hepatology Department, Fundación Santa Fe de Bogotá, School of Medicine, Universidad de los Andes, Bogotá, Colombia; ^2^Fundación Santa Fe de Bogotá, Bogotá, Colombia; ^3^Pathology Department, Hospital Universitari Vall de Hebron, Barcelona, Spain; ^4^GI/Endoscopy Unit, Institut de Malalties Digestives Metaboliques, Hospital Clinic, University of Barcelona, Barcelona, Spain; ^5^Head of Subdirección de Estudios Clínicos y Epidemiología Clínica (SECEC), Fundación Santa Fe de Bogotá, Bogotá, Colombia

## Abstract

**Background:**

Gastroesophageal reflux disease (GERD) is the most frequent chronic gastrointestinal disorder. It is defined as a condition developed when the reflux of gastric contents causes troublesome symptoms (heartburn and regurgitation). This requires adequate treatment since it can lead to long-term complications including esophagus adenocarcinoma. Proton pump inhibitors (PPI) are generally used to treat GERD due to their high-security profile and efficiency on most patients. However, recurrent reflux despite initial treatment is frequent. *N*-of-1 trial is a study that allows the identification of the best treatment for each patient. The objective of this study is to compare the efficacy of standard dose with double dosage of esomeprazole, to improve the GERD symptoms in a single patient.

**Methods:**

A single-patient trial, placebo-controlled, randomized, double-blind, was carried out from September 25th, 2012, to April 26th, 2013. It included one outpatient at the gastroenterology service in a fourth-level hospital, diagnosed with nonerosive reflux disease (NERD). Yet, his symptoms were heartburn and reflux, and his endoscopic results were normal esophageal mucosa, without hiatal hernia, though pathological pH values. A no-obese male without any tobacco or alcohol usage received esomeprazole 40 mg/day and 40 mg/bid for 24 weeks. A standardized gastroesophageal reflux disease questionnaire (GerdQ) was used weekly to evaluate symptom frequency and severity. The consumption of 90% of the capsules was considered as an adequate treatment adherence. D'agostino–Pearson and Wilcoxon test were used to determine normal or nonnormal distribution and compare both treatments, respectively, both with a significant statistical difference of *p* < 0.05.

**Results:**

The patient completed the study with 96% of adherence. The double dosage of esomeprazole did not improve the control of symptoms compared with the standard dosage. Mean symptomatic score was 9.5±0.5 and 10.2±0.6 for each treatment, respectively (*p* > 0.05).

**Conclusion:**

There was no significant improvement in the patient GERD symptoms increasing the dose of oral esomeprazole during the 6 months of study. *N*-of-1 trials in chronic pathologies including GERD are recommended due to their potential value as systematic methods that evaluate therapies without strong scientific evidence.

## 1. Introduction

The gastroesophageal reflux disease (GERD) is the most frequent chronic gastrointestinal disorder in the United States and Europe [1]. According to a recent systematic review, its prevalence in the USA is 18.1%–27.8% [2, 3]. The Montreal consensus defined GERD as a condition that develops when the reflux of stomach contents causes troublesome symptoms and/or complications [4]. Heartburn, known as retrosternal burning, and regurgitation are highly specific symptoms for GERD [5]; these should be present at least weekly as they have been associated with a clinically meaningful quality of life impairment [6]. This entity requires adequate treatment since it can lead to long-term complications such as Barrett esophagus and/or esophagus adenocarcinoma [7].

Proton pump inhibitors (PPI) are generally used to treat patients diagnosed with GERD due to their high-security profile and efficiency on most of the patients [8]. However, a recent systematic review indicates that 17–32% of the patients show recurrent reflux after the initial treatment [9]; between 10–40% of patients with GERD fail to respond symptomatically, either partially or completely, to a standard-dose proton pump inhibitor; these usually have functional heartburn [10]. According to several studies, alternative therapeutic options are considered for those patients who partially respond to the initial PPI treatment [8, 11, 12]. This includes increasing the dose of PPI (i.e., doubling the dose), changing to a different PPI, or adding a supplementary treatment such as the antagonists of the histamine-2 receptors or prokinetic agents [13]. Thus, these treatment options are not clearly established in the literature [14]. In clinical practice, the GERD symptoms regularly persist after the initial PPI treatment, leading physicians to consider the use of PPI double-dose [8].

There is a current controversy between clinical trials and expert opinions whether doubling PPI dosage is indeed clinically useful when treating patients with uncontrolled symptoms after using a standard dose treatment [15–18]. Besides, there is no strong scientific evidence that supports such alternative for heartburn resolution in either erosive esophagitis or nonerosive reflux disease [19]. Patients with GERD usually require a continue therapy, either continuous, at demand, or intermittent [20]. Additionally, GERD has heterogeneous recurrent clinical expressions and many patients frequently experience symptomatic setbacks, which could be easily self-evaluated [21–23].

Several noninvasive strategies have been developed to assess GERD symptoms, such as questionnaires. These are used as screening tools since they are low cost and can be self-administered [3]. The most common questionnaires are Carlsson–Dent, ReQuest, and GerdQ [24]. The GerdQ was developed from 3 validated questionnaires that assess different aspects of GERD: reflux disease questionnaire, gastrointestinal symptom rating scale, and the gastroesophageal reflux disease impact scale [25, 26]. It consists of 6 questions related to symptoms and the impact of the disease (sensitivity of 65% and specificity of 71% for GERD diagnosis), permitting the follow-up of treatments on long-term patient's symptoms [27]. The reason why this score was used in this study, in such scenario, is because a clinical trial with a unique patient becomes a relevant option.

*N*-of-1 trials, also known as individual-patient trials or single-patient trials, are double-blinded, multiple-crossover, comparative trials of treatment effect [28]. In this study, the participant is treated with two or more treatments on multiple occasions during the study, allowing the identification of the best treatment for each patient [29]. It is a very useful experimental design for pathologies such as GERD allowing the efficacy of the individual therapeutic interventions in this condition; it could be routinely used in the clinical practice to optimize the PPI treatment for each patient [30, 31, 32].

The objective of this study is to compare the efficacy of standard dose treatment with PPI (esomeprazole 40 mg/day) compared with double dosage (esomeprazole 40 mg/bid). It will be presented as a randomized-controlled single-patient trial been able to compare two different dosage of PPI validating the use of double PPI dose to improve the GERD symptoms in patients with suboptimal therapeutic response to standard doses of PPI.

## 2. Methods

### 2.1. Trial Design

A single-patient trial, placebo-controlled, randomized, double-blind with 12 pairs of treatment periods was performed as outpatient at the gastroenterology unit of the Hospital Universitario Fundación Santa Fe de Bogotá, Colombia. The study was carried out for 24 weeks (from September 25th, 2012 to April 26th, 2013). The study was approved by the ethics committee from the Hospital Universitario Fundación Santa Fé de Bogotá (HUFSFB), and the protocol followed the Helsinki Declaration. Informed consent was signed by the participant before starting the study.

### 2.2. Eligibility Criteria

Patients with the following criteria were considered eligible: (a) age 18–70 years; (b) heartburn and/or acid regurgitation on ≥2 days/week for ≥2 months before the study; (c) partial therapeutic response to a standard dose of PPIs on ≥2 months of treatment; (d) impedance-pH monitoring positive for esophageal acid reflux, DeMeester score greater than 14, absence of alkaline reflux, and appropriate esophageal emptying; and (e) mental status allowing us to understand the protocol.

### 2.3. Exclusion Criteria

The participants with the following criteria were not selected or excluded from this study: (a) a history of allergies to the medication used in this study; (b) serious systemic disease, pregnancy, lactation, alarm features, motility disorders, or GERD complications; (c) concomitant use of other PPIs, histamine H2-receptor antagonists, prokinetic drugs, herbal medicines, vitamins, or minerals two weeks before the study; and (d) history of esophageal or gastric surgery, Schatzki ring, achalasia, esophageal motility disorders, esophageal candidiasis, or toxic eosinophilic esophagitis.

### 2.4. Interventions and Treatments

**Figure 1:**
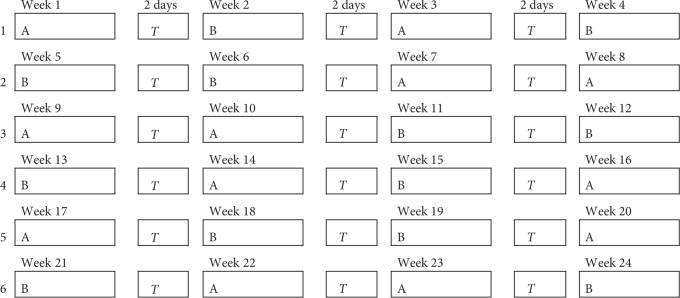
Randomization schedule for one patient receiving 12 pairs (*n* = 24) of treatment, each consisting of one week of treatment A and one week of treatment B. Abbreviations: A, treatment A (esomeprazole 40 mg AM and 40 mg PM); B, treatment B (esomeprazole 40 mg AM and placebo PM); *T*, washout time.

Patients who presented GERD symptoms were referred to confirm the diagnosis and evaluate if they complied with the other requirements to be part of the study, being exempt from the exclusion criteria. The selected patient received two treatments: (A) esomeprazole 40 mg in the morning and night 30 minutes before meal or (B) esomeprazole 40 mg in the morning and placebo at night 30 minutes before meal. Six different determinations of treatment were evaluated and 12 pairs of periods of one week per period (24 weeks) were carried out. Each period was separated by a washout period of 2 days using alginate 10 ml every 12 hours, adding up for a total of 29 weeks of treatment (see Figure 1).

SPSS was used to randomize the sequence of treatment. The medical researchers and the patient were both blind to this randomized distribution. A pharmaceutical laboratory provided the medication in 24 boxes, labeled consecutively from week 1 through 24. Each box contained two blisters identified as “AM” and “PM,” and each one of the blisters had 7 identical capsules in texture, color, and taste. The patient was instructed to take the morning (AM) dose 30 minutes before breakfast and the night (PM) dose approximately 12 hours later.

### 2.5. Measurements during the Study

The efficacy analysis of the treatment in this study was based on weekly follow-up evaluations with the GerdQ questionnaire (Table 1). The adherence to treatment was corroborated by performing a weekly follow-up by asking the patient to register the time they took the medication every day. Additionally, the participant had to return the unused medication to count the number of nonused capsules. We defined an adequate adherence to the treatments if the patient consumed more than 90% of the capsules.

### 2.6. Statistical Analysis

The scores for each pair of treatment periods were weighed. Exploratory data analysis is applied to describe the sample. D'agostino–Pearson test was used to determine if the total scores per treatment were of normal or nonnormal distribution. Wilcoxon allowed a comparison between both treatments. ANOVA random factorial II was applied since the treatments were assigned in a double-blind randomized study design, covering all the possibilities of combination of schemes. Finally, Tuckey HSD and box plot were used to compare the significant differences of the treatment schemes [32, 33]. The entire significance tests were performed taking two tails with a CI of 95% and we considered a significant statistical difference for values of *p* < 0.05.

## 3. Results

This study included a nonobese 58-year-old man with no history of tobacco or alcohol consumption and a body mass index of 26 kg/m^2^. His endoscopic findings were normal both for the esophageal mucosa and esophagogastric junction (EGJ) anatomy. He completed the 12 scheduled randomized pairs of treatments, and the adherence was confirmed to be 96% of the treatment.

The treatment distribution and the scores on the GerdQ questionnaire obtained are shown in Table 2. Symptomatic control was similar during both treatments, and scores of heartburns, regurgitation, stomach pain, nausea, difficulty sleeping due to heartburns or regurgitation, and rescue antacid use were also comparable for esomeprazole 40 mg/day or 40 mg/bid.

**Figure 2:**
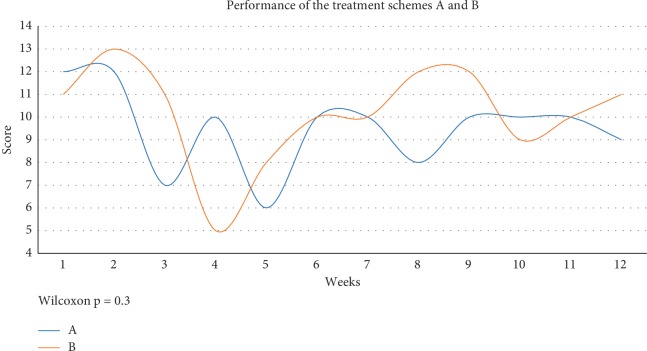
Performance of the treatment schemes A and B according to the total score of the GerdQ scale in the 12 weeks of treatment. Abbreviations: A: treatment A (esomeprazole 400 mg AM and 40 mg PM); B: treatment B (esomeprazole 40 mg AM and placebo PM).

Mean control (±SD) symptoms by treatment A were found to be 9.5±0.5 and the mean control symptoms by treatment B were 10.2±0.6, measured on the GerdQ questionnaire as a high likelihood of having GERD. Mean frequency and severity of each symptom did not significantly decrease over time (*p*=0.30078) (Table 3). The behaviors between the two treatment schemes A and B in the 24 weeks of the study had no significant difference (Figure 2). Thus, ANOVA random factorial II showed a significant difference of symptomatology between the months of study (according to the combinations handled month by month). However, there was no significant difference between the two treatments or in the treatment schedule/month relationship.

**Figure 3:**
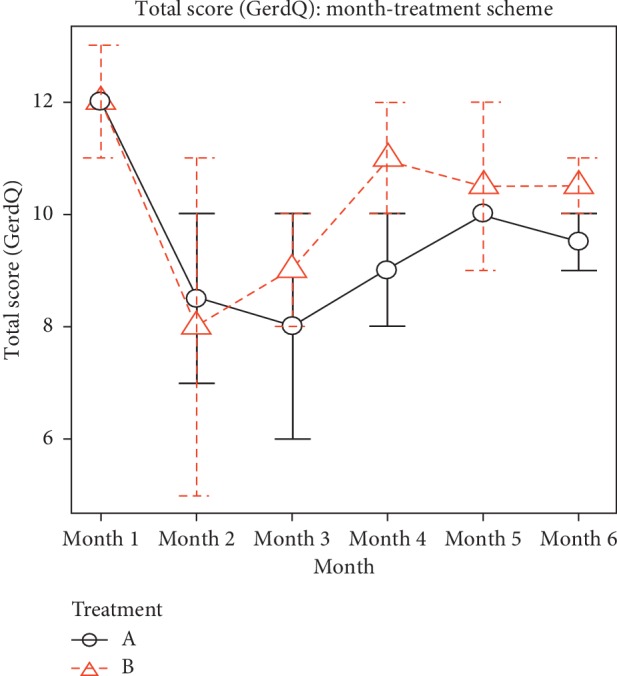
Measurement of total scale (GerdQ) per month of treatment: comparison between schemes A and B. (Tuckey HSD). Abbreviations: A: treatment A (esomeprazole 40 mg AM and 40 mg PM); B: treatment B (esomeprazole 40 mg AM and placebo PM).

Since there was a significant difference in the ANOVA random factorial II, Tuckey HSD and box plot tests were performed to compare the drug schemes with each variable of the scale used and the total monthly score. In Figure 3, scheme B showed a significant difference compared with the total score (months 1, 3, and 6). However, scheme A did not present significant differences in the months evaluated.

## 4. Discussion

Normally randomized controlled trials (RTCs) are the gold standard for evidence-based practice; however, this provides a treatment for an average of patients in a trial [34]. *N*-of-1 trial is used as a very promising tool for patient-centered outcomes research (PCOR) [35]. This type of study is adequate for evaluating long-term treatments for chronic conditions, and it is not suitable for acute conditions or diseases. The following are required for single-patient trials: a stable response to treatment, rapid onset of treatment effect, and negligible expected adverse effects [36]. Therefore, it is a very useful experimental design for pathologies such as GERD allowing the efficacy of the individual therapeutic interventions in this condition.

PPIs are widely prescribed for patients with GERD since they are one of the most potent inhibitors of gastric acid secretion available [32], thanks to their effectiveness in treating heartburn and regurgitation symptoms [5]. However, there is no enough scientific evidence that supports doubling the dose of PPIs to improve symptomatic control, compared with the standard dose [14]. The findings of this study confirm showing that doubling the dose of esomeprazole from 40 mg/day to 40 mg/bid does not improve symptomatic control in a patient with GERD.

A significant number of patients in the world treated with PPIs show a partial response to the treatment due to heterogeneous character of the illness. PPIs are widely prescribed; thus, up to 50–70% of these are either unnecessary or inappropriately prescribed, approximately 113 millions of formulations per year with near 13 billions of dollars in annual sales [37, 38]. Therefore, the relevance of this study is highlighted; it is important for practitioners to identify patients with a complete response compared to partial or no-response to treatment [5].

In this *N*-of-1 clinical design, increasing the dose of PPI did not show an improvement of GERD symptoms as the punctuations of GerdQ questionnaire for both treatments were similar. There was no significant improvement in the average frequency and severity of symptoms during the 6 months of study. Regarding additional studies, some clinical trials report an enhanced efficacy when the PPI dose is duplicated; however, these results contradict other similar studies which show no difference between the efficacy for standard versus double dose of PPI. This implies contradictions between different groups of patients and a large variability of results for different studies.

Cochrane review suggests that doubling the dose of PPI is associated with larger healing rates for erosive esophagitis [8]. However, there is no clear dose-symptomatic response relation for PPIs in erosive esophagitis or NERD [16]. In Thjodleifsson et al.'s study, similar results were obtained; a multicenter, parallel-group, randomized, double-blind trial reported no statistically significant difference in the efficacy rabeprazole 10 mg versus 20 mg in the for a maintenance treatment for GERD [18]. In a multicenter, randomized, double-blind, parallel-group study, analogous results were obtained; Talley et al. showed esomeprazole 20 mg was superior to placebo as an on-demand treatment for GERD, though a larger dose of 40 mg did not give any clinical benefit [39]. Finally, in Fass et al.'s study, patients who did not show symptomatic improvement after an initial therapy using 30 mg of lansoprazole once a day were randomized and separated into two groups for consequent therapy using either a double dose of lansoprazole or 40 mg esomeprazole once a day; no statistically significant difference was found [19].

In controversy to the previously discussed studies, an open-label, randomized, six-way crossover study by Wilder-Smith et al. [17] showed an improvement over acid control in patients with GERD when increasing the dose of oral esomeprazole (20 mg, 40 mg, and 80 mg) and pantoprazole (40 mg and 80 mg). Another similar study reports a significant symptomatic improvement in pediatric population when using 20 versus 40 mg of pantoprazole [40]. Finally, a third open-label, single-center, randomized, six-way crossover study over 40 healthy subjects receiving esomeprazole 20, 40, and 80 mg and lansoprazole 15, 30, and 60 mg once daily for 5 days showed that increasing the dose of esomeprazole and lansoprazole improved significantly the control of acids [41].

The results of this study suggest that considering healthy lifestyles and healthy patients could have a low impact over the partial response to PPI treatment albeit the data available in the literature about the influence of lifestyle over reflux symptoms are inconsistent [42–45]. Moreover, it has been implied that adherence to treatment problems could play a role in the partial response to PPIs, yet this was not observed in our study. In Ruigómez et al. study [46], females tend to be more susceptible to a partial response to PPI therapy, but neither age, overweight, obesity, tobacco, nor alcohol usage were associated with an increased risk towards a partial response to PPIs. Likewise, polimedicated patients, those who show anxiety or depression signs, those with an initial diagnose of esophagus cancer or severe GERD, presented a higher risk of not responding completely to the PPI-based therapy.

This *N*-of-1 trial could be useful to avoid unnecessary clinical conducts like duplicating the dose of PPI in many patients diagnosed with GERD. The aim of this clinical *N*-of-1 trial is to provide scientific evidence that may lead to eliminating unnecessary doubling of PPI dosage. Thus, we recommend more *N*-of-1 trials in the GERD field due to their potential value as systematic methods that evaluate therapies without strong scientific evidence; performing *N*-of-1 trials, contemplating other GERD phenotypes, and testing different PPI dosages would be interesting for further studies. Furthermore, *N*-of-1 trials can be used for comparing a wide range of therapies for many gastroenterological diseases, while providing individual-focused outcomes, the combined *N*-of-1 trial design offers promising bridging between research and clinical practice.

This study has several strengths: 1. the doses investigated were the same as those used in clinical practice; 2. the study was performed in a patient in whom our approach was clearly applicable; 3. at the end of the study, the patient reported a better understanding of his illness (GERD) due to his involvement in the results of the study; 4. any possible correlation between 7-day periods was eliminated by including a two-day washout period; and 5. the large number of observation periods (12 pairs of treatment periods, sample size) increased the statistical significance of the results and reduced the risk for false inferences or shadowing.

The limitations of this study were 1. being a single-patient trial, the findings can be nonrepresentative for other groups of patients; 2. a healthy patient was selected compared to regular clinical practice patients with obesity, different comorbidities, polypharmacy, usage of tobacco or alcohol, and so on; 3. a close follow-up process was performed during the 24 weeks of treatment, including weekly medical visits and phone calls to obtain adequate adherence to the therapy; and 4. a potentially memory bias could be present given the high-frequency reply to the GerdQ every week; one month is the time frame which ensures a good balance between education of recall bias and having appropriate information on outcome measures [47].

Evidence-based medicine suggests that clinicians should rely on the results of randomized controlled trials (RCTs) over groups of patients; however, even when relevant RCTs generate a probable answer, their results may not apply to a given individual patient. Thus, *N*-of-1 trials are an opportunity for tailor treatments to individual patients considering that these trials are at the top of the hierarchy in terms of strength of evidence for treatment decisions [48]. Also, patients appear to benefit from the participation in such trials given their improved sense of involvement and control over their treatments, as well as an increase in care focused on the individual, which is not commonly available in the routines of clinical practice.

Therefore, investing and increasing the frequency of use of *N*-of-1 trials is expected to be a positive factor in progressing towards patient-centered care and shared decision-making in clinical practice [49]. Clinicians can incorporate the *N*-of-1 RCTs into their medical practice for determining the optimum treatment of a given individual patient. Lastly, the *N*-of-1 approach clearly has the potential of improving the quality of medical care in patient with chronic diseases. This is definitely an innovative patient-centered approach to drug management [50].

In conclusion, there was no significant improvement in the patient GERD symptoms increasing the dose of oral esomeprazole during the 6 months of study. *N*-of-1 trials in chronic pathologies including GERD are recommended due to their potential value as systematic methods that evaluate therapies without strong scientific evidence.

## Figures and Tables

**Table 1:** The GerdQ questionnaire respondent enters the frequency scores after reflecting his/her symptoms over the previous week [27].

Questions/frequency	0 days	1 day	2-3 days	4–7 days
1. How often did you have a burning feeling behind your breastbone (heartburn)?	0	1	2	3

2. How often did you have stomach contents (liquid or food) moving upwards to your throat or mouth (regurgitation)?	0	1	2	3

3. How often did you have pain in the center of the upper stomach?	3	2	1	0

4. How often did you have nausea?	3	2	1	0

5. How often did you have difficulty getting a good night of sleep because of your heartburn and/or regurgitation?	0	1	2	3

6. How often did you take additional medications for your heartburn and/or regurgitation other than those your physician told you to take (such as Gaviscon, Tums, Rolaids, and Maalox)?	0	1	2	3

Scoring information: GerdQ score was calculated as the sum of these scores, giving a total score ranging from 0 to 18. Those with a score of 8 or more have a high likelihood of having gastroesophageal reflux disease (GERD), and those with less than 8 have low or no likelihood.

**Table 2:** GerdQ ^†^ score for the subject participating in individually evaluable GERD ^‡^ single-patient trial.

Week	Treatment	Total symptoms (score)	1. Heartburn (score)	2. Regurgitation (score)	3. Stomach pain (score)	4. Nausea (score)	5. Difficulty sleeping with heartburn/regurgitation	6. Antacid use
1	A	12	2	3	0	1	3	3
2	B	11	2	3	0	1	3	2
3	A	12	0	3	1	2	3	3
4	B	13	2	3	1	3	2	2
5	B	11	2	2	1	3	2	1
6	B	5	0	1	1	3	0	0
7	A	7	2	2	0	2	1	0
8	A	10	0	2	3	3	2	0
9	A	6	0	0	3	3	0	0
10	A	10	0	2	3	3	1	1
11	B	8	1	1	3	3	0	0
12	B	10	0	2	3	3	0	2
13	B	10	0	2	3	3	0	2
14	A	10	0	2	3	3	0	2
15	B	12	0	2	3	3	2	2
16	A	8	0	2	3	3	0	0
17	A	10	0	2	3	3	0	2
18	B	12	0	2	3	3	2	2
19	B	9	0	2	3	3	0	1
20	A	10	0	2	3	3	0	2
21	B	10	0	0	3	3	2	2
22	A	10	0	2	3	3	0	2
23	A	9	0	1	3	3	0	2
24	B	11	0	2	3	3	1	2

A: treatment A (esomeprazole 40 mg AM and 40 mg PM); B: treatment B (esomeprazole 40 mg AM and placebo PM). ^†^GerdQ: gastroesophageal reflux disease questionnaire. ^‡^GERD: gastroesophageal reflux disease.

**Table 3:** Results for treatment comparisons of the *N*-of-1 trial (12 pairs of treatments, 24 weeks).

	Weeks of treatment	Mean-controlled symptoms (GerdQ)	Standard deviation	D'Agostino–Pearson	Wilcoxon test *p* value
Treatment A (±SD)	12	9.50	0.5	0.598	0.30078
Treatment B (±SD)	12	10.17	0.6	0.035	

^†^GerdQ: gastroesophageal reflux disease questionnaire.

## Data Availability

The results of the application of the GerdQ questionnaire data used to support the findings of this study are included within the article. Any other data used to support the findings of this study are available from the corresponding author upon request.

## Abbreviations

PPIs: Proton pump inhibitors
GERD: Gastroesophageal reflux disease
CI: Confidence interval
RCTs: Randomized clinical trials
HUFSFB: Hospital Universitario Fundación Santa Fe de Bogotá.
